# The core microbiome of cultured Pacific oyster spat is affected by age but not mortality

**DOI:** 10.1128/spectrum.00031-24

**Published:** 2024-08-20

**Authors:** Anna Cho, Jan F. Finke, Kevin X. Zhong, Amy M. Chan, Rob Saunders, Angela Schulze, Snehal Warne, Kristina M. Miller, Curtis A. Suttle

**Affiliations:** 1 Department of Microbiology and Immunology, The University of British Columbia, Vancouver, British Columbia, Canada; 2 Department of Botany, The University of British Columbia, Vancouver, British Columbia, Canada; 3 Hakai Institute, Heriot Bay, British Columbia, Canada; 4 Department of Earth, Ocean and Atmospheric Sciences, The University of British Columbia, Vancouver, British Columbia, Canada; 5 RKS Labs, Parksville, British Columbia, Canada; 6 Pacific Biological Station, Fisheries and Oceans Canada, Nanaimo, Canada; 7 Institute for the Oceans and Fisheries, The University of British Columbia, Vancouver, British Columbia, Canada; Connecticut Agricultural Experiment Station, New Haven, Connecticut, USA

**Keywords:** metamorphosis, core microbiome, aquaculture, Pacific oyster, spat, *Crassostrea gigas*, *Magallana gigas*, amplicon sequencing, 16S rRNA

## Abstract

The Pacific oyster is the most widely cultured shellfish worldwide, but production has been affected by mortality events, including in hatcheries that supply the seed for growers. Several pathogens cause disease in oysters, but in many cases, mortality events cannot be attributed to a single agent and appear to be multifactorial, involving environmental variables and microbial interactions. As an organism’s microbiome can provide resilience against pathogens and environmental stressors, we investigated the microbiomes in cohorts of freshly settled oyster spat, some of which experienced notable mortality. Deep sequencing of 16S rRNA gene fragments did not show a significant difference among the microbiomes of cohorts experiencing different mortality levels, but revealed a characteristic core microbiome comprising 74 taxa. Irrespective of mortality, the relative abundance of taxa in the core microbiomes changed significantly as the spat aged, yet remained distinct from the microbial community in the surrounding water. The core microbiome was dominated by bacteria in the families *Rhodobacteraceae*, *Nitrosomonadaceae*, *Flavobacteriaceae, Pirellulaeceae,* and *Saprospiraceae*. Within these families, 14 taxa designated as the “Hard-Core Microbiome” were indicative of changes in the core microbiome as the spat aged. The variability in diversity and richness of the core taxa decreased with age, implying niche occupation. As well, there was exchange of microbes with surrounding water during development of the core microbiome. The shift in the core microbiome demonstrates the dynamic nature of the microbiome as oyster spat age.

**IMPORTANCE:**

The Pacific oyster (*Magallana gigas*, also known as *Crassostrea gigas*) is the most widely cultivated shellfish and is important to the economy of many coastal communities. However, high mortality of spat during the first few days following metamorphosis can affect the seed supply to oyster growers. Here, we show that the microbiome composition of recently settled oyster spat experiencing low or high mortality was not significantly different. Instead, development of the core microbiome was associated with spat aging and was partially driven by dispersal through the water. These findings imply the importance of early-stage rearing conditions for spat microbiome development in aquaculture facilities. Furthermore, shellfish growers could gain information about the developmental state of the oyster spat microbiome by assessing key taxa. Additionally, the study provides a baseline microbiome for future hypothesis testing and potential probiotic applications on developing spat.

## INTRODUCTION

Native to eastern Asia, the Pacific oyster (*Magallana gigas*, also known as *Crassostrea gigas*) is the most widely cultivated oyster worldwide (1). Despite its resilience to a broad range of environmental conditions, mass mortality events have been reported globally, including France (2), Australia (3), California, USA (4), and British Columbia, Canada (5). Mortality events occur at all life stages in both natural-spawning reefs (6) and farms (7–10). However, mortality events affecting both larvae or post-metamorphosis stage spat in hatcheries would be of special concern as they can limit the availability seed to growers. Consequently, growers in the Pacific Northwest (11, 12) and Alaska (13) that rely heavily on hatchery spat have faced economic challenges. Despite numerous studies (2–5, 7–10, 14, 15), the causes of many mortality events remain elusive.

Studies have attributed the effects of ocean acidification (15, 16), temperature (17), farming location and practices (5), and emerging pathogens (18) as potential causes of mortality. More recent studies have shown that Pacific oyster mortality syndrome, prevalent in adult oysters, is a polymicrobial disease involving a myriad of environmental factors (19), oyster genetics (20–22) and multiple biotic agents such as Ostreoid Herpes Virus (OsHV) (14, 23, 24) and opportunistic bacteria (25).

There is evidence that the emergence of disease can be linked to changes in the host core microbiome (26–30). These reports lead to our initial hypothesis that the microbiomes between moribund (high mortality) and healthy (low mortality) spat are different, and our aim to establish a “core” microbiome of oyster spat.

As demonstrated by microbiome studies of other aquatic organisms, the functional influence of a core microbiome can be direct, as exemplified by bacterial symbionts of bobtail squid directly involved in metabolic processing (31). The influence can also be indirect as some core microbes only occur at a certain life stage or environmental condition, in which their presence is correlated to resistance to pathogens and better adaptability (32, 33). These core microbiomes can in turn differ among tissues, life stages, species, and growth environments. Moreover, there is evidence from other marine invertebrates such as corals and shellfish that a disturbed microbiome is associated with the host being susceptible to pathogens (28–30), some which switch from a commensal or transient lifestyle. The state at which a disturbed microbiome becomes the cause of disease has been described as a pathobiome (26, 27). These reports led us to hypothesize that there is a “core” microbiome that is altered in spat experiencing high mortality.

The core microbiome of oysters has been characterized from different life stages, tissues, sizes, farm locations, and broodstock using a variety of sequencing methods (17, 34–41). However, most studies have focused on microbiome dynamics in adult oysters under specific stressors (17, 35, 37) or candidate pathogens (8, 14, 24, 42, 43), and have not considered recently settled spat. However, the development of the spat microbiome is likely critical as they transform from a planktonic to a benthic lifestyle. This was demonstrated in an experiment in which immune genes and epigenetic expressions differed between mature larvae exposed to seawater enriched with naturally occurring non-pathogenic microbes and ones exposed to hatchery-standard UV-treated seawater (44). Furthermore, the larvae with early microbial exposure had enhanced survival when challenged with OsHV-1. Similarly, age-related (45) and metamorphosis-related microbiome shifts in insects (46, 47), amphibians (32), and fish (48) have been linked to changes in metabolic processes and defense against pathogens.

In the present study, the microbiomes of early-stage oyster spat were analyzed over 43 days, during which cohorts experienced different rates of mortality. We observed a core microbiome in spat that was distinct from that of the surrounding water and significantly changed in the relative abundance of taxa as the spat aged. At the same time, the variability in richness and diversity of members of the core microbiome decreased. However, the composition of the spat microbiome did not correlate significantly with mortality. These results demonstrate that the core microbiome of freshly settled Pacific oyster spat changes with age, but not with mortality.

## RESULTS

### Data overview

A total of 47 samples, comprised of 39 oyster spat samples from nine cohorts, and eight water samples were collected from May to October 2014. Spat cohorts were reared at temperature, salinity, and pH-adjusted conditions (Table 1) according to industry standards. We define a cohort as a single settlement of newly post-metamorphosed spat (>310 µm) in a nursery tank. The age of spat ranged from 0 to 43 days post-metamorphosis. Some cohorts experienced up to 99% mortality within the first 2 weeks of the spat setting in the nursery tanks, with the observed daily mortality (ODM) peaking at 80% for the high-mortality cohorts and 25% for the low-mortality cohorts (Table 1). As it is usual to have some mortality in developing spat, we defined “high-mortality” cohorts as ones that experienced >50% mortality and “low-mortality” as ones with <50% mortality based on the standard of the hatchery; however, low-mortality cohorts experienced a maximum of 25% daily mortality, far less than the 80% daily mortality in the high-mortality cohorts.

**TABLE 1:** Growth parameters and mortality status of oyster spat samples^*a*^

Cohort	Year	Month	Day	Age	ODM	Mort.	Temp.	Sal.	Alk.	pH
							°C	0/00	mg/L	
1	2014	May	21	5	10	Low	17.8	31	3.12	8.52
1	2014	May	28	12	25	Low	16.4	30	4.16	8.46
1	2014	June	4	19	0	Low	20.2	25	3.76	8.37
1	2014	June	11	26	0	Low	18.1	24	3.55	8.14
5	2014	Jul	9	4	1	High	21.8	22	4.25	8.08
5	2014	Jul	11	6	80	High	/	/	/	/
5	2014	Jul	16	11	15	High	23.3	25	4.27	8.16
5	2014	Jul	23	17	0	High	19.4	22	4.2	8.58
13	2014	Jul	9	4	10	High	21.8	22	4.25	8.08
13	2014	Jul	12	7	80	High	/	/	/	/
13	2014	Jul	16	11	1	High	23.3	25	4.27	8.16
13	2014	Jul	23	18	1	High	19.4	21	4.2	8.58
13	2014	Jul	30	25	5	High	24.3	25	2.74	8.09
14	2014	Jul	23	7	5	Low	18.1	22	4.17	8.4
14	2014	Jul	30	14	1	Low	24	25	2.92	8.12
14	2014	Aug	6	21	5	Low	21.1	24	3.71	8.25
14	2014	Aug	13	28	1	Low	18.7	26	3.87	8.17
14	2014	Aug	20	35	5	Low	20.6	25	6.26	8.34
15	2014	Jul	30	3	1	Low	24.4	23	2.78	8.19
15	2014	Aug	6	10	1	Low	20.9	25	3.44	8.4
15	2014	Aug	15	17	1	Low	19.2	25	3.21	8.59
16	2014	Aug	6	0	0	High	20.1	22	3.34	8.33
16	2014	Aug	13	7	80	High	18	26	3.3	8.2
16	2014	Aug	20	14	1	High	20.9	26	6.34	8.48
18	2014	Sep	3	1	0	Low	15.4	26	3.44	8.47
18	2014	Sep	10	8	10	Low	21.3	26	3.12	8.46
18	2014	Sep	17	15	5	Low	13.4	29	3.15	8.78
18	2014	Sep	24	22	2	Low	13.6	29	3.45	8.7
18	2014	Oct	1	29	2	Low	12.5	30	3.31	8.76
18	2014	Oct	8	36	0	Low	13.1	30	3.02	8.01
18	2014	Oct	15	43	1	Low	11.3	30	2.89	7.98
19	2014	Sep	10	5	80	High	21	26	3.08	8.42
19	2014	Sep	17	12	20	High	13.4	29	3.15	8.78
19	2014	Sep	24	19	10	High	13.6	29	3.45	8.7
19	2014	Oct	1	26	1	High	12.5	30	3.31	8.76
19	2014	Oct	8	33	1	High	13.1	30	3.02	8.01
19	2014	Oct	15	40	0	High	11.3	30	2.89	7.98
20	2014	Sep	24	0	0	Low	12.5	29	3.41	8.89
20	2014	Oct	1	7	5	Low	12.4	30	3.28	8.85
20	2014	Oct	8	14	1	Low	13.1	30	3.02	8.1
20	2014	Oct	15	21	1	Low	11.3	30	2.89	7.98

^*a*^ Columns are the cohort number, sampling date and age in days post settling of spat, ODM rates, mortality status (Mort.), temperature (Temp.), salinity (Sal.), alkalinity (Alk.), and pH as measured in tanks. Unavailable data are indicated by “/”.

**Fig 1:**
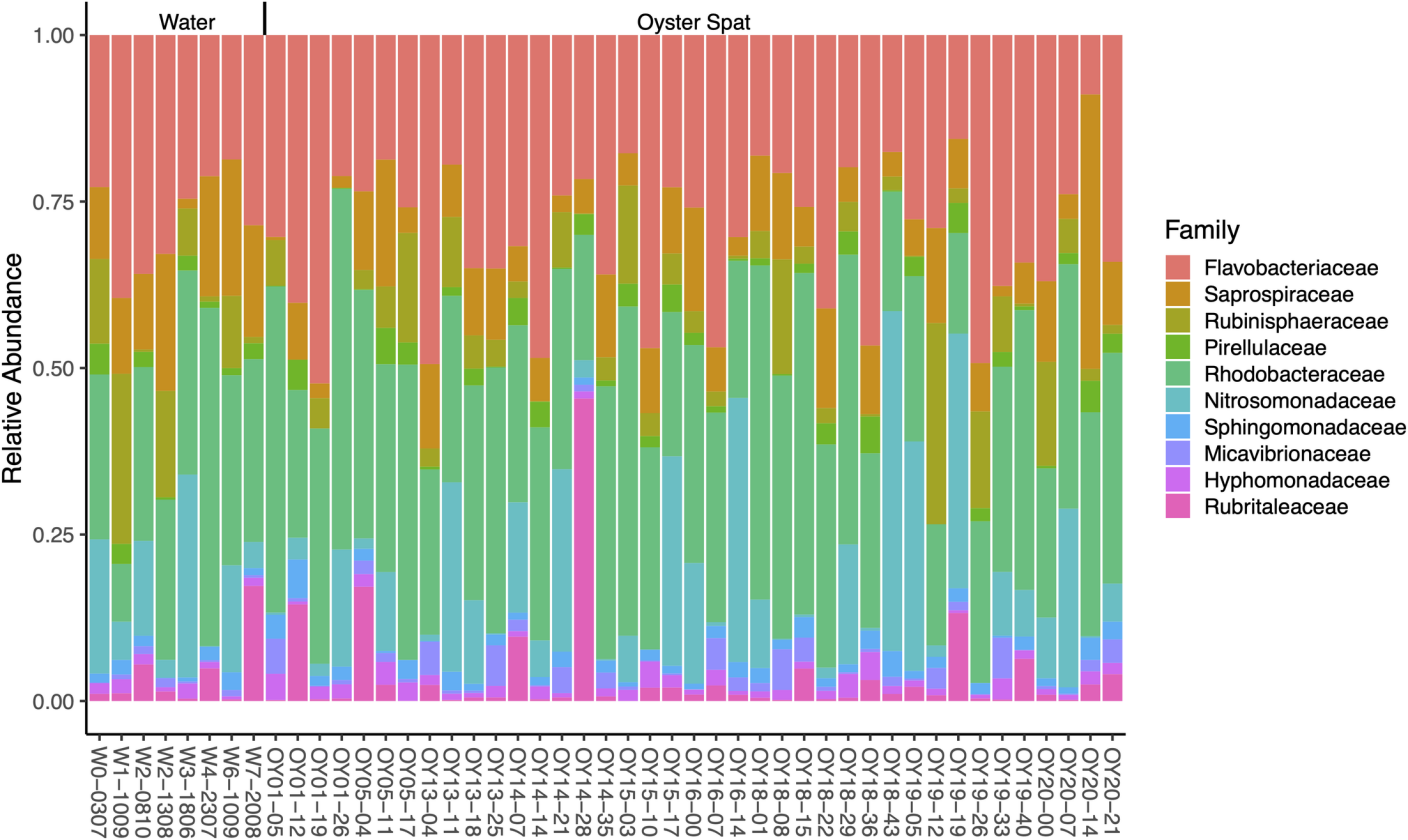
Overview of the relative abundances of the 10 most abundant families of bacteria in the combined sequencing data based on 16S rRNA gene sequences. Water samples are labeled “W” followed by tank number and sample date (ddmm). Spat samples are labeled “OY” followed by cohort number and the number of days post settlement.

Sequencing of 16S rRNA gene V4–V5 amplicons yielded 1,373,696 reads. The combined oyster and water samples produced 21,506 biological sequence variants, which were analyzed as unclustered OTUs (operational taxonomic units). On average, 27,450 reads per sample were generated. The reads were mapped to the OTUs to produce a frequency matrix and were rarefied to the minimum number of reads per sample (16,881). Taxonomic annotation based on SILVA (v.132) resulted in 91.8% of OTUs being annotated to phylum, 87.3% to class, 77.8% to order, 73.1% to family, 39.5% to genus, and 13.2% to species. Collapsing taxa at the family level resulted in 257 families, with *Rhodobacteraceae* (*Pseudomonadota*) and *Flavobacteriaceae*, *Nitrosomonadaceae* and *Saprospiraceae* (*Bacteroidota*)*,* and *Rubinisphaeracea* and *Pirellulaeceae* (*Planctomycetota*) being dominant. There were no clear differences between water and oyster samples in the relative abundances of the top 10 bacterial families (Fig. 1). Other abundant families (data not shown) included *Cyanobiaceae (Cyanobacteria*) and *Vibrionaceae (Pseudomonadota*). Also, there was no significant difference in diversity between the microbiomes of “low-mortality” and “high-mortality” cohorts (Table S1). A constrained distance-based redundancy analysis (dbRDA) showed weak separation between microbiomes of low- and high-mortality samples, and among daily mortalities (Fig. S1), but mortality only explained 2.9% of the microbiome variation and was not significant (*P*-value 0.279).

**Fig 2:**
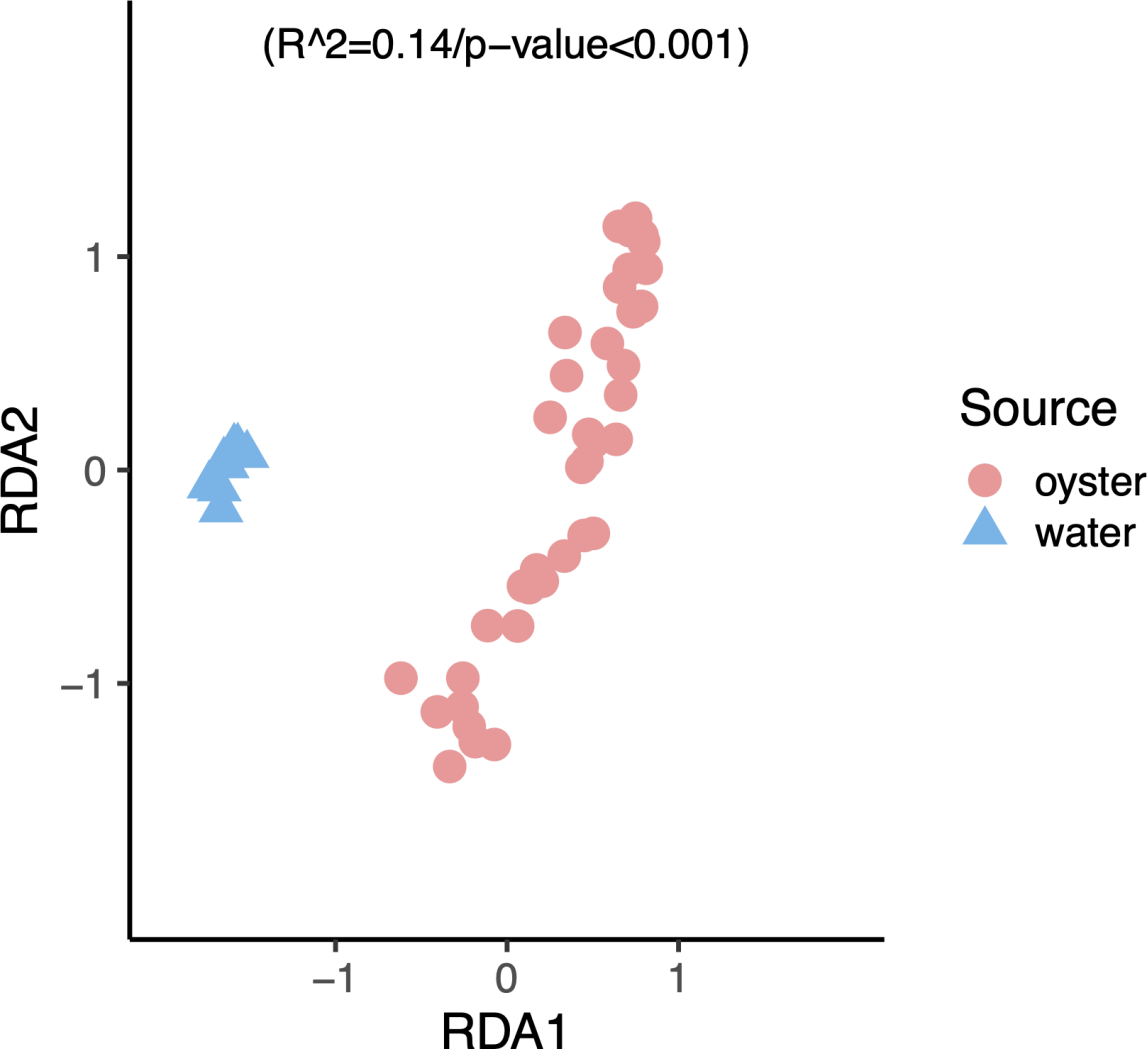
Constrained dbRDA of the community composition of water and oyster samples against the sample source; results of the PERMANOVA are shown. Blue triangles are water samples; red circles are oyster samples.

To assess the dissimilarity of microbial communities between oyster and water samples for all taxa, a constrained dbRDA of oyster samples against the sample source (i.e., tank water) was performed. The dbRDA (Fig. 2) shows that water samples (blue triangles) were tightly clustered and clearly separated from oyster samples (red circles). Although there was a lot of variation among the oyster samples, they were significantly different from the water samples (*P*-value <0.001) based on a permutational multivariate analysis of variance (PERMANOVA).

### **The core microbiome of** Pacific oyster spat

**Fig 3:**
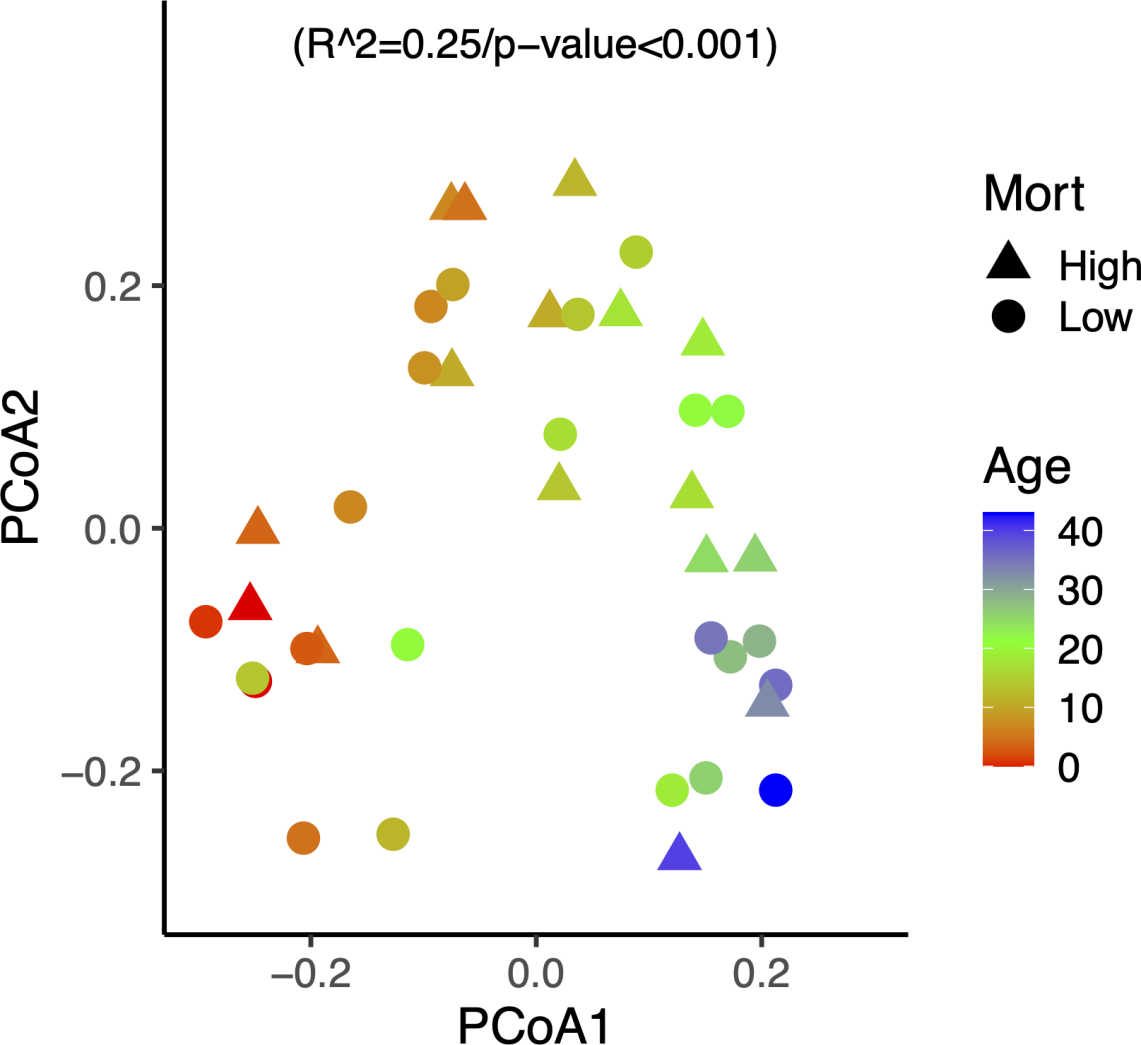
Principal Coordinate Analysis (PCoA, unconstrained dbRDA) of the community composition of core OTUs. Shapes indicate whether cohorts experienced low <50% /d (circles) or high >50% /d (triangles) mortality. Color indicates the age in days post settlement. Results of a subsequent PERMANOVA of community distance against sample age are shown.

To identify taxa constituting the core microbiome of spat, we selected OTUs present in more than 50% of all oyster samples, and that were differentially represented in oyster samples compared to water samples. Both conditions had to be fulfilled for an OTU to be part of the core microbiome. This approach identified 74 core OTUs that were predominant in the families *Rhodobacteraceae, Flavobacteriaceae*, and *Nitrosomonadaceae*, but also included taxa in the *Saprospiraceae*, *Haliaceae*, *Bdellovibrionaceae,* and unassigned OTUs (summarized in Table S2). An unconstrained dbRDA of the core microbiome composition in oyster samples showed a pattern of shifting community similarity with spat age (Fig. 3). While some outliers are observed, there is a gradual transition of the core microbiome as the spat aged, which was confirmed to be significant in a subsequent PERMANOVA (*P*-value <0.001) of core microbiome similarity against age. Notably, the dbRDA of the core taxa did not show a correlation between the core microbiome and mortality rates. In comparison, an unconstrained dbRDA of the whole microbiome also showed variation with age, but the subsequent PERMANOVA against age explained less of the variation than for the core microbiome (Fig. S2).

To identify representative OTUs from the core microbiome that best describe the age-related microbiome shift, a balance-tree analysis was used. The analysis calculates balance values for samples based on the difference in ratio of selected OTUs with regard to age. The analysis identified five OTUs belonging to taxa within the *Rhodobacteraceae, Flavobacteriaceae,* and *Nitrosomonadaceae*, which were used to calculate balance values for each sample (Table S3). The combination of the selected OTUs resulted in a significant linear correlation (Fig. S3) between their relative abundance in each sample and age (*R*^2^ = 0.9266; *P*-value <2e − 16).

### Evolution of the core microbiome with age

**Fig 4:**
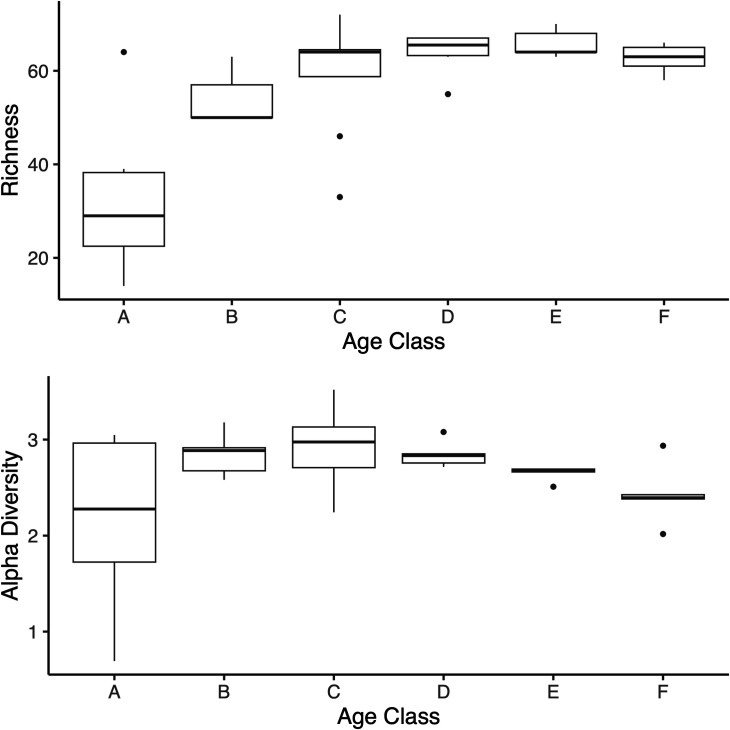
Core microbiome diversity indices for spat age classes; indices were calculated based on the community compositions of samples per age class. Top graph depicts OTU richness; bottom graph shows alpha diversity, age classes (days post settlement), A (0 to 4), B (5 to 11), C (12 to 17), D (18 to 23), E (24 to 39), and F (≥30), which had respective sample sizes (*n*) of 6, 9, 8, 6, 5, and 5. Box plots describe the minimum and maximum values, lower and upper quartiles and median, outliers.

**Fig 5:**
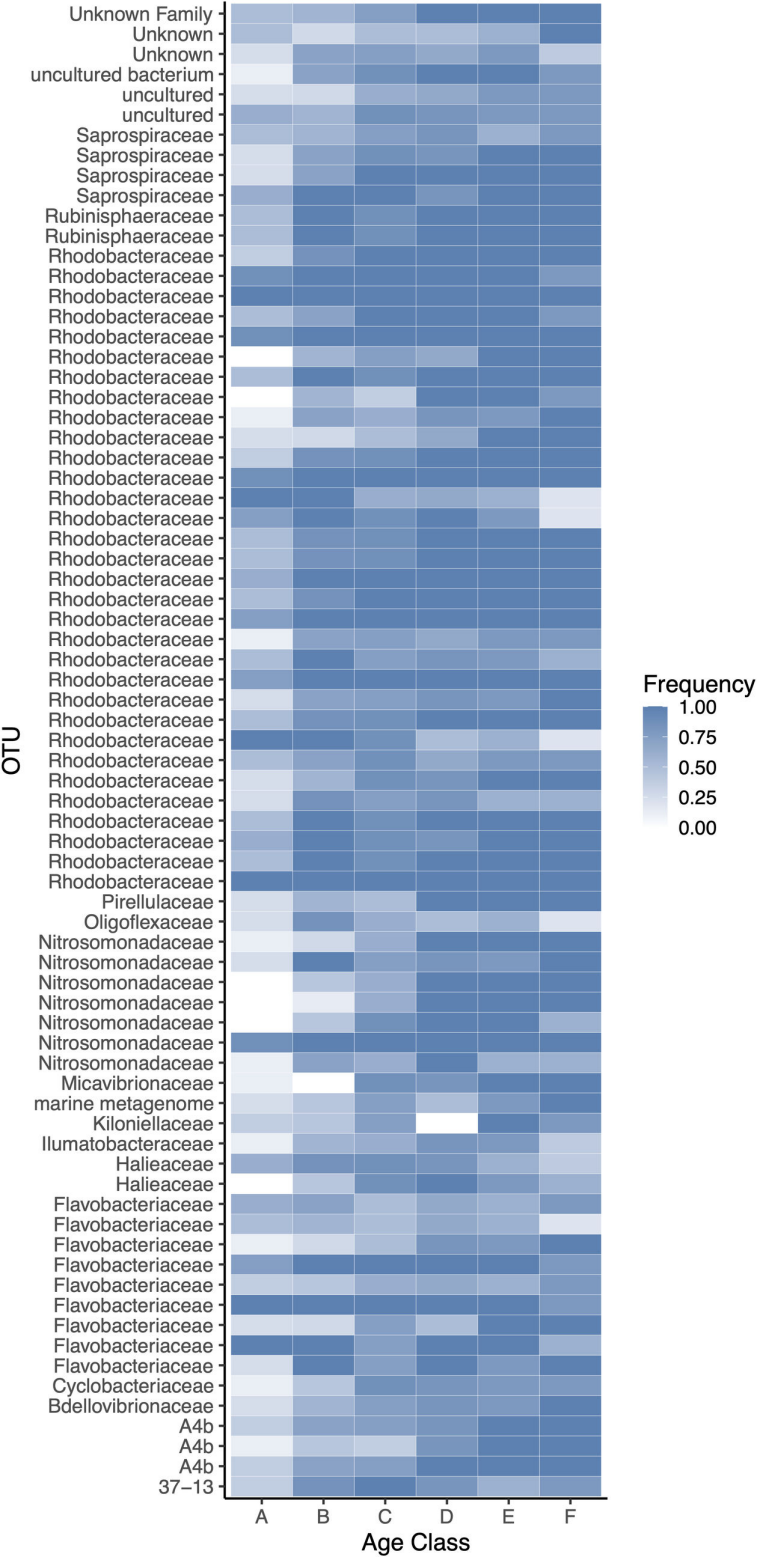
Heat map of the frequency of core OTUs across samples per age class. Color tone describes frequency per age class; white is absence and dark blue is presence. OTUs are defined at the genus level, but family annotation is labeled.

To better understand how the core microbiome in oyster spat changed with age, spat samples were categorized into six age classes (days since post-metamorphosis), A (0 to 5), B (6 to 11), C (12 to 17), D (18 to 23), E (24 to 29), and F (≥30), which had respective sample sizes (*n*) of 8, 7, 8, 6, 5, and 5. Generally, the richness of the core microbiome increased with increasing age, more so in age classes A to C than D to F. Meanwhile, the variability in richness within age classes decreased (Fig. 4). Similarly, alpha diversity increased from A to C, while average diversity and especially the variability in diversity dropped in classes D to F. The frequency of core OTUs across samples in each age class mirrored the diversity indices (Fig. 5). Most core OTUs occurred less frequently across samples in age classes A to C, but were more prevalent in D, E, and F. However, some OTUs showed the opposite trend; two OTUs in the *Rhodobacteraceae* had a high frequency in A, but were almost absent in F. Overall, 11 OTUs, mainly in the *Nitrosomonadaceae* and *Rhodobacteraceae*, showed a significant increase and two OTUs (*Rhodobacteraceae*) showed a significant decrease in linear models with progressing age class (Table S3).

Comparing early age classes A to C against late age classes D to F in an indicator species analysis (49) uncovered eight significant indicator species. Two OTUs in the *Rhodobacteraceae* were indicators for the early age classes, while three OTUs in the *Nitrosomonadaceae* and one each in the *Rhodobacteraceae, Pirellulaceae* and A4b (phylum *Chloroflexi*) were indicator species for the later age classes (Table S3). The combined analyses were consistent for 14 OTUs that had significant linear correlations to age. Several of these OTUs increased in frequency with age, were indicator taxa for the older age classes, and were key taxa in the balance model. Accordingly, the OTUs that decreased in frequency with age were indicator taxa for younger age classes (Table S3).

**Fig 6:**
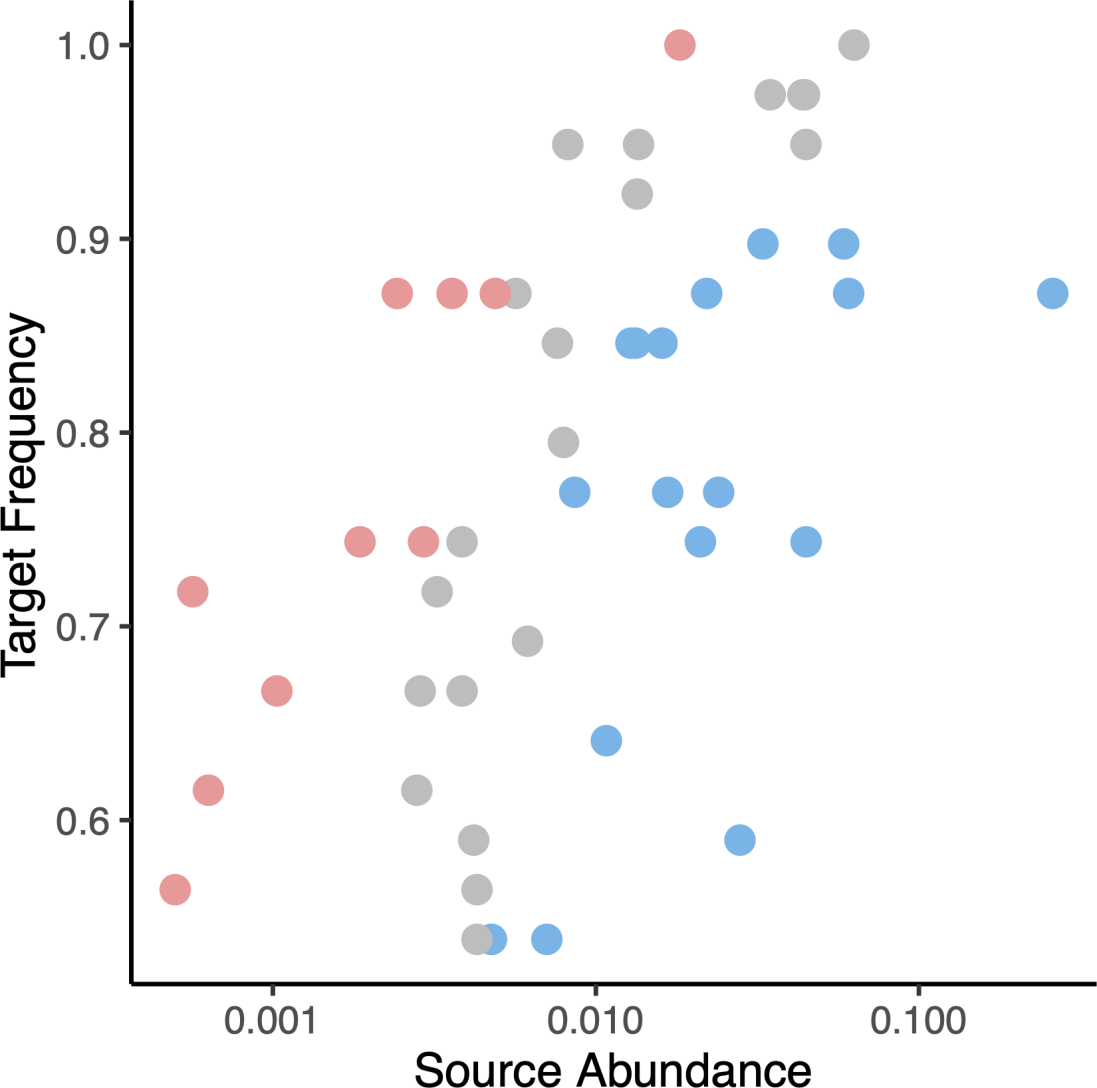
Mean abundance in water communities (source abundance) and frequency across oyster spat samples (target frequency) for OTUs included in the neutral community model analysis. Colors indicate the dispersion mode: red for OTUs overrepresented in oyster samples; blue for OTUs overrepresented in water samples; gray for OTUs where the observed frequency matched the predicted frequency in the neutral community model.

Finally, a neutral community model (50) was applied to explore the dispersion of core OTUs from surrounding water to spat. This analysis could only be performed on a subset of 47 core OTUs that were present in spat and water samples. The predicted frequency of the 47 OTUs in the oyster communities was calculated based on their mean relative abundance in water samples. Twenty OTUs (gray circles) with observed frequencies within the 95% CI of their predicted frequencies were designated to be neutrally dispersed from water to oyster spat (Fig. 6). Ten OTUs with higher observed than predicted frequencies (red circles) were designated to be overrepresented in oysters, and 17 OTUs with lower observed frequencies than predicted (blue circles) were designated to be underrepresented. The neutrally dispersed OTUs were predominantly 11 OTUs in the *Rhodobacteraceae*, 2 in the *Saprospiraceae*, and 1 in each of the *Nitrosomonadaceae* and *Flavobacteriaceae* (summarized in Table S4). Six of the overrepresented OTUs were in the *Rhodobacteracea*, while the underrepresented OTUs were predominantly in the *Flavobacteriaceae* and *Rhodobacteracea*.

## DISCUSSION

We explored changes in the microbiome of recently settled Pacific oyster spat that experienced a range of mortality. Samples were collected to reflect microbiome development in recently settled spat grown under standard aquaculture practices (11). The composition of the spat microbiome was not correlated with mortality; however, we revealed a core microbiome that differed significantly from that in the water. Moreover, the core microbiome was defined by a relatively small number of taxa that changed in relative abundance with age, and were dispersed through water. These observations and their implications are elaborated on below.

In total, 21,506 OTUs were identified, providing high taxonomic resolution; 73% of these were classified at the family level for subsequent analysis. The most abundant OTUs were in the *Rhodobacteraceae, Flavobacteriaceae*, *Nitrosomonadaceae,* and *Saprospiraceae*. Bacteria in these families are common in aquatic environments and belong to the major phyla *Bacteroidetes* and *Pseudomonadota* (*Proteobacteria*)*,* which also occur in the hemolymph of adult and juvenile oysters (17, 35, 37, 51, 52), as well as in marine waters (53). Additionally, members of the *Planctomycetota* and *Cyanobacteria* were abundant, and are also commonly found in seawater (53). Overall, at the family level, the dominant bacteria were consistently present across oyster spat and water samples. Nonetheless, given that the dbRDA and PERMANOVA analyses showed significant differences between the OTUs of the oyster and water microbiomes, we could use the OTU data from the water as a baseline to identify the core microbes characteristic of oysters. Differences between the composition of the microbial communities in oysters and the surrounding water resemble observations from previous studies (52, 54) implicating niche occupation of oyster-associated microbes. However, our findings differ from those in King et al. (39), in that we did not find a significant correlation between microbiome community and mortality. This can be due to different causes of mortality or oyster ages.

While there is no single definition of a core microbiome, we followed the broad definition that the core microbiome is comprised of microbial taxa that are consistently associated with given ecological or biological habitats (55, 56). Consequently, the core microbiome was based on taxa that were more abundant in spat than in the adjacent water, and which had a frequency over 50% across spat samples. In a study on coral core microbiomes, the number of taxa and their relative abundance drastically increased when the frequency cut-off values were lowered from 90% to 50% (57). This suggests that a higher percentage cut-off value would mask important shifts of the core microbiome over time. Since, in our study, the microbial composition was not significantly different between “low-mortality” and “high-mortality” spat samples, we decided to include all spat samples when defining the core microbiome. It should be noted that sample processing and sequencing methods can affect the interpretation of microbiome analysis (34).

Based on the described criteria, 74 out of 21,506 OTUs were selected as part of the core microbiome; primarily, these OTUs belonged to the families *Rhodobacteraceae, Nitrosomonadaceae,* and *Flavobacteriaceae*. Bacteria in these families were also found in mature and juvenile oyster microbiomes (17, 51, and reviewed in 54) and 7-month-old spat, with the relative abundance of some families varying, depending on the size of the spat (41); the latter study also identified a comparable number of 97 core taxa (41). Differences in the composition and abundances of the core taxa among these studies are likely related to differences in oyster species and tissues, sampling and processing methods, rearing location and conditions, targeted 16S rRNA region, and age of the oysters (41, 51, 52, 58). Interestingly, several core taxa are related to those identified in the seaweed microbiome, which consisted of 24 core taxa in the *Rhodobacteraceae*, *Flavobacteriaceae,* and *Saprospiraceae* for different host life stages (59), and we speculate that these taxa may be better adapted to fast-changing environments.

In the present study, the relative abundance of taxa in the core microbiome was significantly correlated to spat age (Fig. 3). Similar shifts in microbiome composition with age have been documented in oysters from different life stages (36), and a recent study on the spat microbiome under experimental temperature and pCO_2_ stress also found a change of the microbiome composition with age (60). A correlation between the microbiome development and host age has also been documented in corals, insects, and fish (45, 47, 61, 62). Notably, in a separate project on mature oyster microbiomes, no correlation with age was apparent (40), suggesting that early developing oyster spat provide quickly changing habitats for microbes. The taxonomic shift in the core microbiomes was further quantified by balance-tree analysis; sample age explained 93% of the occurrence of five reference OTUs in the *Rhodobacteraceae, Flavobacteriaceae,* and *Nitrosomonadaceae*. Changes of the relative abundance of *Flavobacteriaceae* have also been induced by temperature and infection stress in oysters (17). Similarly, Trabal-Fernandez et al. (36) observed in oysters that, at the genus level, bacteria in the *Pseudomonadota* varied between post-larval and adult stages. The observed shifts in core microbiome composition within families can be explained by successful niche occupation of phylogenetically and ecologically similar taxa.

We also investigated the dynamics of temporal shifts in the core microbiome. For most core OTUs, the frequency increased with progressing age classes; the 11 OTUs with significant increase in frequency may play a key role in oyster development. Similar patterns of microbiome changes with host development were observed in fish (48, 61, 62), insects (46, 47), but especially corals (45). Williams et al. (45) and Yan et al. (62) also observed that most OTUs increased in their relative abundance with early coral and fish microbiome development, while few OTUs were lost and replaced in older spat. The indicator species analysis further supported this observation with six OTUs that were characteristic for later age classes (D, E, and F) and only two *Rhodobacteracea* OTUs that were indicator taxa for the early age classes (A, B, and C). The two late age classes indicator taxa, *Proteobacteria* and *Planctomycetes,* were also found in mature oysters in a separate analysis (40). Furthermore, the core microbiome development was reflected by an initial increase of taxa richness and diversity with age but subsequent decrease in the variability of core taxa. Again, Williams et al. (45) observed a similar increase in microbiome richness in developing corals, while Yan et al. (62) showed decrease in alpha diversity of the gut microbiome during metamorphosis of fish larvae.

Finally, the neutral model analysis of core OTUs revealed the dispersal of taxa from rearing water to oyster microbiomes (50). The 20 *Rhodobacteraceae* OTUs fitting the neutral model were likely dispersed from water to the oysters as they aged. Of the OTUs not fitting the neutral model, the 10 overrepresented OTUs appear to be selected for in the oysters, while 17 underrepresented OTUs appear to be selected against. Dispersed and selected for OTUs are likely permanent members of the core microbiome, but the OTUs that are selected against are more likely to be lost from the core microbiome during aging. However, the OTUs that do not fit the neutral model may also be the result of stochastic processes of dispersal and losses (63). These findings show that dispersal and selection processes are shaping the oyster core microbiome as has been seen in other marine environments (64). Furthermore, studies on coral microbiomes also suggest that microbes are dispersed through water (65), as well as vertically transmitted (66).

Altogether, bacteria in the families *Rhodobacteraceae, Flavobacteriaceae, Nitrosomonadaceae*, and *Pirellulaceae* and the phylum *Chloroflexota* (family label A4B) have a significant role in the core microbiome development across analyses. The family *Rhodobacteraceae* comprises diverse groups of marine bacteria, from phototrophs and chemoheterotrophs involved in sulfur and carbon cycling to symbionts of other marine organisms (67, 68). Previous studies also found that members of the *Rhodobacteraceae* can originate from algal feedstock in aquaculture (30). The families *Nitrosomonadaceae* (*Pseudomonadota*) and *Pirellulaceae* (*Planctomycetota*) comprise groups of ammonia-oxidizing bacteria, and have been associated with deep-sea octocorals (*Paramuricea placomus*) (69) and kelp (*Macrocystis pyrifera*) (70). *Nitrosomonadaceae* have also been recorded in coral microbiomes (71, 72). These nitrifying taxa could be ubiquitous in marine filter feeders and important in biogeochemical cycles (70, 73), or simply opportunists occupying ammonium-rich systems. The families *Flavobacteriaceae* and *Vibrionaceae* include common aerobic marine microbes, as well as putative pathogens (30, 74–76). Interestingly, the phylum *Chloroflexota* which encompasses a metabolically diverse group of bacteria has been reported in the gut of other adult oyster species (35, 77), and is one of the core taxa in sponge microbiomes (78), suggesting they may be a common taxon in filter-feeding marine animals. Furthermore, many of the core bacteria are dispersed through the water column, hinting at the role of water in shaping the developing microbiome and a potential avenue for developing probiotic applications in rearing water (30). Fourteen OTUs from these bacterial families appear to have an outsized role in the core microbiome development and could thus be considered the “Hard-Core Microbiome.” Assessing these key taxa of the core microbiome can provide shellfish growers with information about the developmental state of the oyster spat microbiome. The roles of the core taxa remain unclear, warranting research on the key metabolically relevant gene expressions with an emphasis on changing filter-feeding behavior and physiology.

## MATERIALS AND METHODS

### Oyster and water samples

Pacific oyster spat and water samples from a commercial oyster hatchery in British Columbia, Canada, were collected over several months in 2014. The broodstock was a collection of naturally spawning Pacific oysters from Discovery Passage, BC, Canada. Spat were grown by industry standards under optimal conditions at 21°C, pH 8.1–8.2, salinity 25 ‰, and 3.3 mg/L alkalinity; intake water was filtered through sand and 5 µm pore-size filters. Tank temperatures were measured using a mercury thermometer, and salinity was measured with a refractometer. Water pH was measured with a glass probe pH meter (Jenco, San Diego, CA) and alkalinity was measured with a HI901 titrator (Hanna Instruments, Smithfield, RI). Spat size ranged between 400 and 1,000 µm, and the percentage mortality was assessed daily using a microscope. Settlements of post-metamorphosed spat (>310 µm) kept in a nursery tank were defined as a cohort. Each week, about 15 to 30 spat per cohort were collected in 2 mL screw-cap cryovials for sequencing of 16 rRNA gene fragments. Daily observed mortality ranged between 10% and 99%; based on the standards of the hatchery, cohorts that experienced >50% mortality were considered as “high mortality,” while <50% mortality was considered “low mortality.” Therefore, we classified the cohorts into two groups based on the degree of the peak mortality observed. Corresponding water samples for 16S rRNA gene sequencing were taken by filtering 2 L of tank water through 0.22 µm pore-size cellulose acetate membranes (Millipore Sigma, Burlington, MA), and storing the membranes at −80°C until processing.

### DNA extraction

Approximately 20 oyster spat were transferred into sterile 1.5 mL microcentrifuge tubes using a sterile spatula or pipette. Using two 3.2 mm diameter chrome steel beads (Bio Spec Products Inc., Bartlesville, OK), the spat were homogenized in a Mixer Mill MM300 (Retsch GmbH, Haan, Germany) for 1 min with a frequency of 1/30 s. At least 2 mg of tissue homogenate from each tube was transferred to a new microcentrifuge tube, and DNA extracted using a DNeasy Blood & Tissue Kit (Qiagen, Hilden, Germany) per manufacture’s protocol, with an added lysozyme (10 mg/mL) lysis step after proteinase K addition. DNA from water samples was extracted using a DNeasy PowerWater Kit (Qiagen, Hilden, Germany) following the manufacturer’s protocol. DNA extracts were quantified with the Qubit dsDNA HS Assay Kit (Life Technologies, Carlsbad, CA).

### 16S rRNA gene amplification, library preparation, and sequencing

The 16S sequencing libraries were prepared following the Illumina MiSeq 16S Metagenomic Preparation Guide using the Nextera XT v2 Kit (Illumina, San Diego, CA), with the following minor modifications. PCR mixtures contained 1 to 10 ng of template DNA in a 25 µL reaction volume. The V4–V5 (515F-926R) hypervariable regions of the small subunit 16S ribosomal RNA coding gene were targeted for amplification with barcoded forward and reverse primers. Conditions for PCR were denaturation for 5 min at 95°C followed by 34 cycles at 95°C for 45 s, 50°C for 45 s, 68°C for 90 s, and a final extension at 68°C for 10 min. The reaction mix contained 1× Q5 Reaction Buffer [New England Biolabs (NEB), Ipswich, MA], 0.4 µM barcoded forward and 0.4 µM reverse primers, 0.1 U Q5 High-Fidelity DNA Polymerase (NEB, Ipswich, MA), 0.8 mM deoxynucleotide triphosphyate (dNTPs), 1× Bovine Serum Albumin (BSA) (100×), and 2.5 mM MgCl_2_. The Index-PCR reaction was performed in a 25 µL volume containing 10 ng DNA, using Nextera XT v2 Index 1 and 2 Primers. The reaction mix contained 1× Q5 Reaction Buffer (NEB, Ipswich, MA), 1.25 µL each of Nextera XT v2 Index 1 and 2 Primer, 0.1 U Q5 High-Fidelity DNA Pol (NEB, Ipswich, MA), 0.4 mM dNTPs, 2 mM MgCl_2_. Amplicon libraries were purified and size-selected using Agencourt AMPure XP Beads (Beckman Coulter Inc., Brea, CA), and quantified with the Qubit dsDNA HS Assay Kit (Life Technologies Inc., Carlsbad, CA). The Index attachment was verified by quantitative PCR using the SsoFast EvaGreen Supermix (Bio-Rad Laboratories Inc., Hercules, CA) and KAPA DNA Standards (KAPA Biosystems, Wilmington, MA) on a C10000 Touch PCR block with a CFX 96W Reaction Module (Bio-Rad Laboratories, Inc., Hercules, CA). Libraries were pooled and gel-purified in a 1.5% agarose gel (1× Tris-Borate-Ethylenediaminetetraacetic acid, TBE) by excising the 560 base-pair (bp) band using a Zymoclean Gel DNA Recovery Kit (Zymo Research Corp., Irvine, CA), following the manufacturer’s instructions. The pooled libraries were validated with a Bioanalyzer High Sensitivity DNA chip (Agilent Technologies, Inc., Santa Clara, CA) and sequenced at the BRC Sequencing Core at The University of British Columbia on a MiSeq platform using 2 × 300 bp paired-end chemistry (Illumina, San Diego, CA).

### Bioinformatic and statistical analysis

Raw read data were demultiplexed followed by removal of primers, low quality reads (Phred < 29) and chimeras using Quantitative Insights into Microbial Ecology (QIIME 2) v.2018.6.0 (79). Forward and reverse reads were merged using the Divisive Amplicon Denoising Algorithm 2 (DADA2) in QIIME 2 (79, 80). The method produced a list of features, each representing a biological sequence variant, generated by the *de novo* error correction method of DADA2. Features were designated as OTUs based on the 99% sequence similarity against SILVA reference database (release v.132) using QIIME2 (79, 81). A sequence similarity tree was generated using *de novo* multiple sequence alignment (MAFFT) (82); divergent OTUs with long branches were searched using the Basic Local Alignment Search Tool (83), and mitochondria, chloroplasts, metazoan, and singletons were filtered from the OTU table. OTU frequency in samples was determined by read recruitment using QIIME2 (79); the frequency matrix was normalized to the lowest number of total reads per sample by rarefication with the VEGAN package (84) in the R environment (85) producing representative microbial communities per sample.

A taxonomic overview was generated by collapsing OTUs at the family level with the corresponding phyla. Microbial community composition was compared between water and oyster samples in a constrained dbRDA using VEGAN; significance of the separation was assessed by a PERMANOVA test. Differential abundance of taxa between water and oyster samples was determined with the DESeq2 package (86) in R. To compare the similarity in community composition among samples, the Bray-Curtis dissimilarity index was calculated and ordinated using an unconstrained dbRDA in VEGAN, with subsequent PERMANOVA test for significance. This is based on shared OTUs that were present in more than 50% of all oyster samples, and were differentially represented in oyster and water microbiomes (see core microbiome). Sample richness (i.e., species richness) and Shannon alpha diversity were assessed based on the core taxa and calculated with the VEGAN package. Taxa with the best linear correlation to age were determined with the Selbal package (87). Indicator taxa for age classes were identified with the Indicspecies package (88, 89). Dispersion of taxa from the water to oysters were tested with a neutral community model based on Sloan et al. (50) in R.

### Core microbiome

Similar to other studies, we defined the core microbiome as shared OTUs that occurred in more than 50% of oyster samples, and which were differentially represented in spat than in the surrounding water (29, 55, 90). The core microbiome was defined based on spat that experienced low or high mortality as there was no significant difference in the microbiome composition between groups. Both criteria had to be fulfilled for an OTU to be considered part of the core microbiome.

## Data Availability

The sequencing data used in this study have been deposited in the NCBI Short Read Archive (SRA) under the accession number PRJNA1032956. All other data supporting the findings of this study are provided in the manuscript or as supplementary files.

## SUPPLEMENTAL MATERIAL

Figure S1 spectrum.00031-24-s0001.tiff Constrained dbRDA of the community composition.

Figure S2 spectrum.00031-24-s0002.tif An unconstrained dbRDA (PCoA) of the whole microbiome.

Figure S3 spectrum.00031-24-s0003.tiff Linear model of the calculated balance values.

Table S1 spectrum.00031-24-s0004.xlsx Pairwise Kruskal-Wallis test.

Table S2 spectrum.00031-24-s0005.xlsx OTUs designated as the core microbiome with normalized abundance values per sample.

Table S3 spectrum.00031-24-s0006.xlsx Linear models with R2, slope, and significance (*P*-value).

Table S4 spectrum.00031-24-s0007.xlsx OTUs included in the neutral community model analysis.

ASM does not own the copyrights to Supplemental Material that may be linked to, or accessed through, an article. The authors have granted ASM a non-exclusive, world-wide license to publish the Supplemental Material files. Please contact the corresponding author directly for reuse.
