# Supplementary figures and images for: The core microbiome of cultured Pacific oyster spat is affected by age but not mortality
Source: Microbiol Spectr. 2024 Aug 20;12(10):e00031-24. doi: 10.1128/spectrum.00031-24 (PMC11448229; doi:10.1128/spectrum.00031-24)

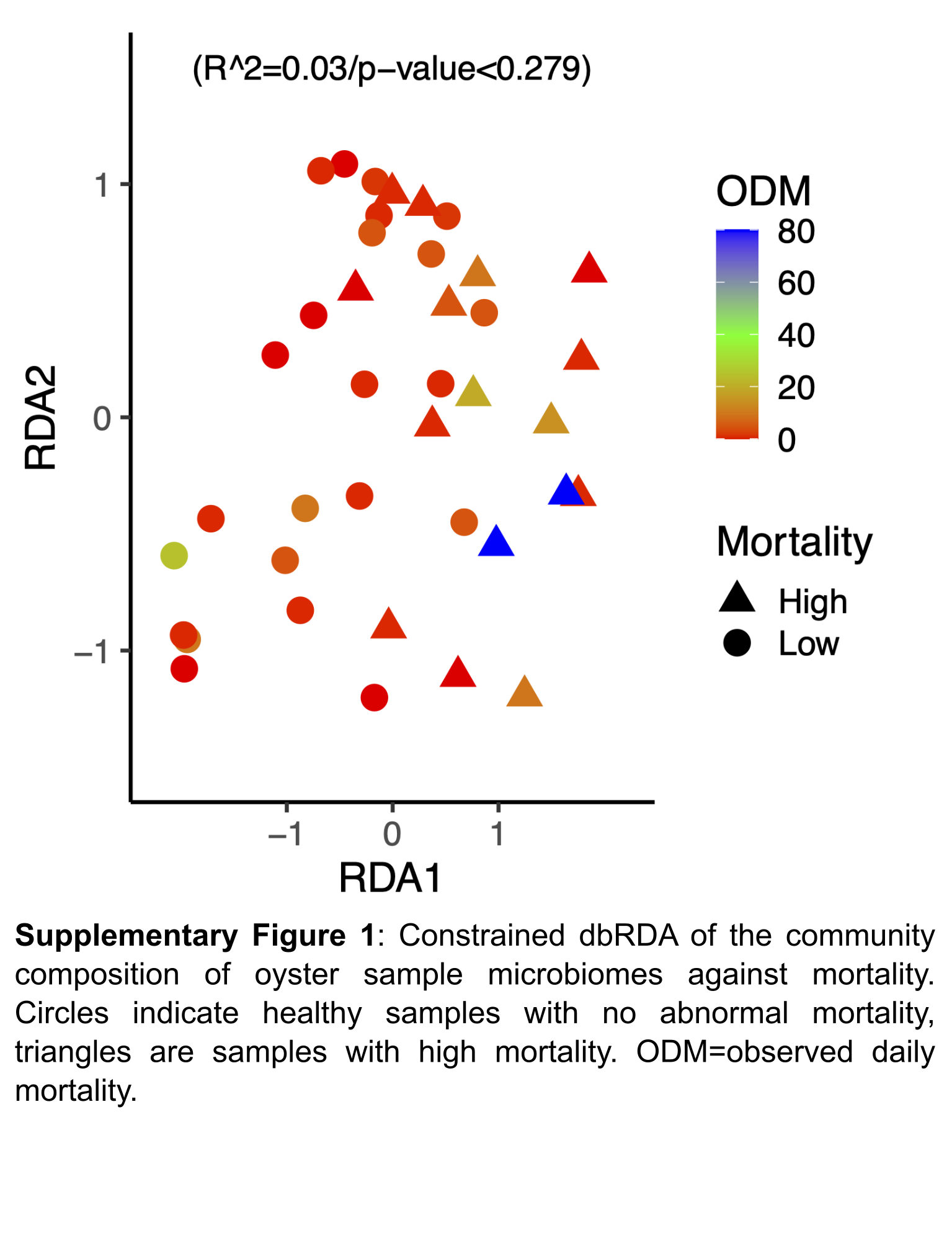

Supplement: Figure S1 — Constrained dbRDA of the community composition. [file spectrum.00031-24-s0001.tiff]

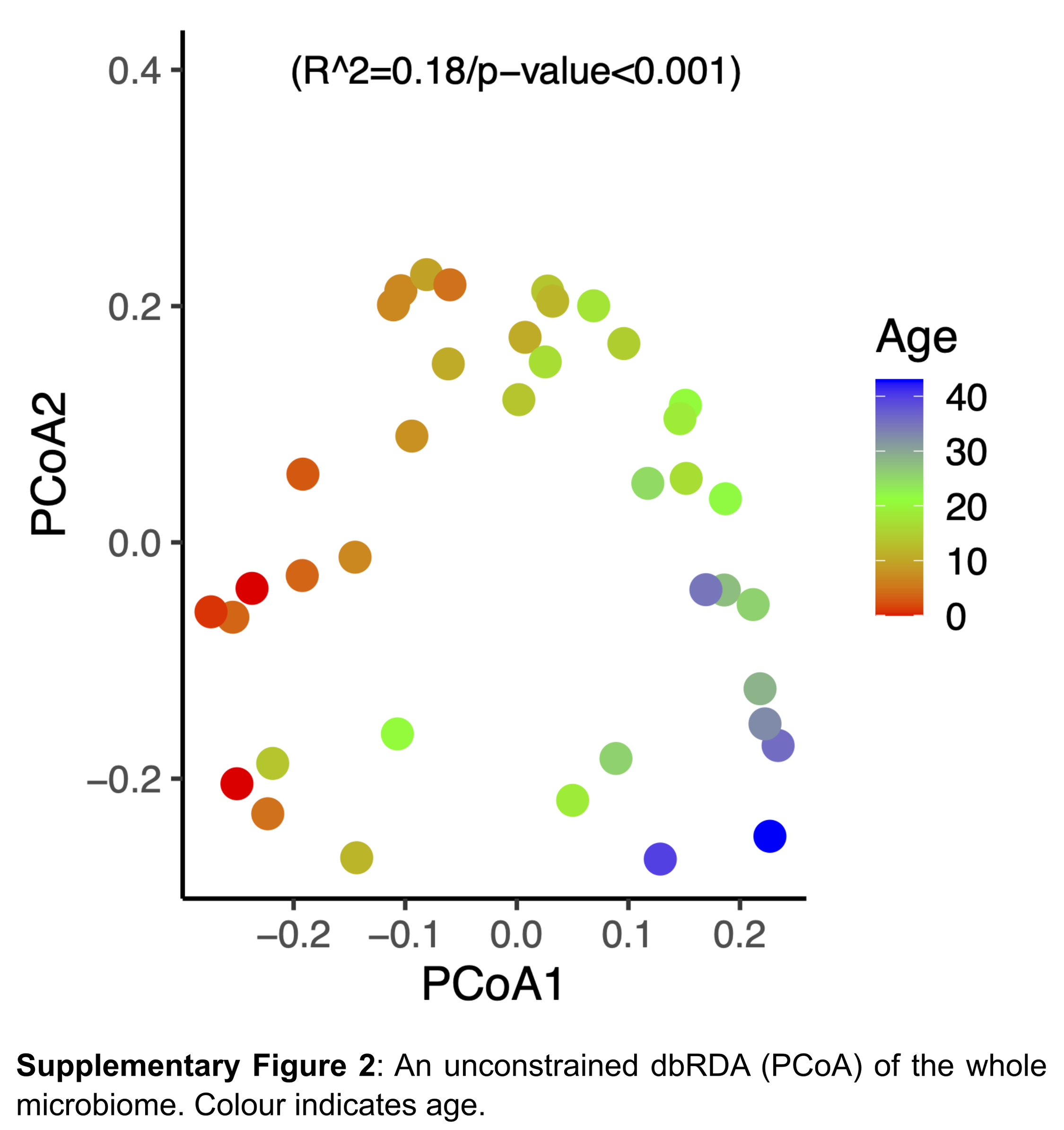

Supplement: Figure S2 — An unconstrained dbRDA (PCoA) of the whole microbiome. [file spectrum.00031-24-s0002.tif]

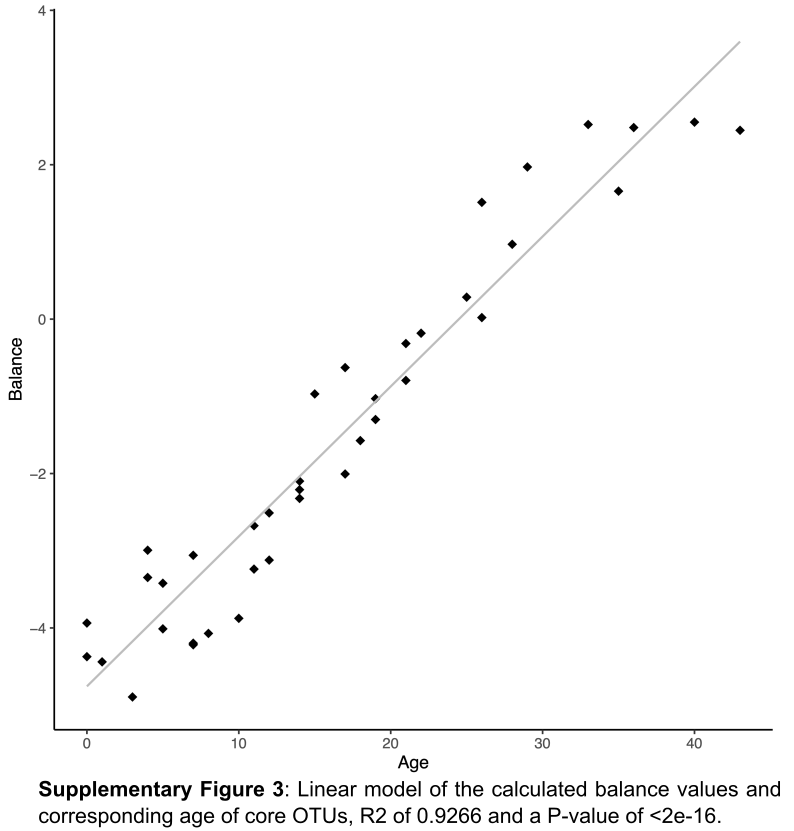

Supplement: Figure S3 — Linear model of the calculated balance values. [file spectrum.00031-24-s0003.tiff]
